# A tabletop exercise approach to global disaster preparedness: insights from Nepal’s first international conference on disaster preparedness and management

**DOI:** 10.3389/fpubh.2024.1400588

**Published:** 2024-06-11

**Authors:** Jay Pandya, Ramu Kharel, Jonathan McMahon, Samjhana Basnet, Samara Soghoian, Binita Pradhan, Bonnie Arquilla

**Affiliations:** ^1^Department of Emergency Medicine, UT Southwestern Medical Center, Dallas, TX, United States; ^2^Department of Emergency Medicine, Warren Alpert Medical School of Brown University, Providence, RI, United States; ^3^Department of Emergency Medicine & Internal Medicine, SUNY Downstate Health Sciences University, Brooklyn, NY, United States; ^4^Department of General Practice and Emergency Medicine, Dhulikhel Hospital, Kathmandu University School of Medical Sciences, Dhulikhel, Nepal; ^5^Department of Emergency Medicine, Bellevue Hospital, New York University Grossman School of Medicine, New York, NY, United States; ^6^Department of General Practice and Emergency Medicine, Kathmandu Medical College, Kathmandu, Nepal

**Keywords:** disaster resiliency building, disaster medicine, health systems strengthening, interdisciplinary education, disaster preparedness, tabletop exercises, developing health systems

## Abstract

Considering recent earthquakes and the COVID-19 pandemic, disaster preparedness has come to the forefront of the public health agenda in Nepal. To strengthen the developing health system, many initiatives are being implemented at different levels of society to build resiliency, one of which is through training and education. The first International Conference on Disaster Preparedness and Management convened in Dhulikhel, Nepal on December 1–3, 2023. It brought together international teaching faculty to help deliver didactic and simulation-based sessions on various topics pertaining to disaster preparedness and management for over 140 Nepali healthcare professionals. This paper focuses on the tabletop exercise-based longitudinal workshop portion of the conference on disaster leadership and communication, delivered by United States-based faculty. It delves into the educational program and curriculum, delivery method, Nepali organizer and US facilitator reflections, and provides recommendations for such future conferences, and adaptation to other settings.

## 1 Introduction

Disasters are events which stress a community’s resources and response capabilities beyond its capacity. One component of the disaster cycle is disaster preparedness, which involves developing operational response plans and conducting trainings, drills, and exercises, to ensure that the people and systems involved are prepared (1). Exercises are frequently used in disaster preparedness efforts to evaluate and practice response policies and skills (2). The tabletop exercise (TTX) is a specific type of discussion-based exercise where a scenario is presented to the participants and as the scenario develops, the participants must discuss their response actions in the confines of the plans and policies in place and their assigned roles and responsibilities (3). It is used with the intended aims of providing and applying a conceptual understanding of disaster policies and procedures, identifying areas of strength and improvement through discussion, and fostering leadership and communication skills (3). TTXs require low costs and low resources, and can be done in a stress-free informal, classroom, or workshop setting. This makes them useful for preparedness efforts, particularly in low resource settings. Guidelines provided by the United States Department of Homeland Security’s Exercise and Evaluation Program (HSEEP) and prior studies have shown that adapting the scenario to the context, characteristics, and settings similar to those that participants are likely to encounter in practice, is crucial for ensuring a meaningful experience for the participants (3–6). Exercises are built around hypothetical or “model” cities or communities, using a technique that is frequently used in emergency management and by the US Federal Emergency Management Agency for training purposes (7, 8).

Nepal, one of the most highly ranked disaster-prone countries globally, adopted the Sendai Framework for Disaster Risk Reduction in 2015 by establishing the National Disaster Risk Reduction Strategic Action Plan (9, 10). This constitutional mandate provided a national disaster management framework, with an incorporated incident command system (ICS) framework, for all levels of the government. However, similar to other developing healthcare systems, the implementation of this national plan is slow to reach the community level (10).

In an effort to fill this implementation gap, a group of Nepali emergency medicine and general practice physicians led by the Nepal-based co-authors of this paper, organized and hosted the First International Conference on Disaster Preparedness and Management in Nepal, at the Dhulikhel Hospital in December 2023. The United States-based faculty group and co-authors of this paper were invited to facilitate the TTX-based longitudinal workshop portion of this conference. Given that this was a major focus of the conference, this paper focuses on its conception and components, and provides a model for adaptation in other developing health systems.

## 2 Methods

### 2.1 Conference

Dhulikhel Hospital hosted the World Academic Council of Emergency Medicine’s (WACEM) section for Crisis and Disaster Medicine to facilitate a Regional Congress for Tabletop Exercise and Communication in Crisis Disaster Medicine (TOPCOM), during this First International Conference on Disaster Preparedness and Management in Nepal. WACEM is a collaborative international council focused on the academics of emergency sciences. WACEM’s section for Crisis and Disaster Medicine has been hosting an annual TOPCOM workshop since 2021, focusing on the use of TTXs to discuss emergency scenarios, test local emergency response plans, and practice communications skills in simulated emergency scenarios (11). Our US-based faculty group was invited to facilitate the TTX workshop under this premise. The conference brought together over 140 Nepali participants and was facilitated by invited international faculty from India, Malaysia, and the United States (US). The mission of the conference was:

*To develop technical and non-technical knowledge, skills, and attitudes during disaster response; to improve disaster response utilizing Hospital ICS implementation; and to improve leadership skills during disaster response. To improve patient and healthcare workers’ safety during disaster, intra or inter-departmental and interhospital communication and coordination skills; and to improve hub and satellite hospital networking* (12).

### 2.2 Site and participant selection

Conference organizers for this inaugural conference are a group of emergency medicine and general practice physicians. They chose Dhulikhel Hospital as the site because of its disaster-prone location, and for logistical convenience. It is an independent, non-profit, non-governmental Kathmandu University-affiliated hospital located 30 kilometers southeast of the capital, Kathmandu. It has been the site of disaster response for earthquakes including the 7.8 magnitude earthquake in 2015, landslides, road traffic accidents, and recent COVID-19 pandemic. It also serves as a hub hospital and tertiary care referral center for its catchment area of 2.5 million people (13).

The conference organizing committee invited participants through targeted selection. Invitations were sent to healthcare personnel involved in all aspects of disaster response and coordination from all 25 hub hospitals in the country, as well as the 11 Kathmandu University-affiliated medical colleges in the Kathmandu Valley. After recruiting invited participants, registration was kept open for all others who were interested. Any English-speaking healthcare worker, over 18 years of age, with an interest in disaster management was eligible to enroll.

The conference was made possible through financial, logistical, and technical support from national and local governmental and non-government agencies, World Health Organization, Nepal Red Cross Society, Rotary Club, Government of Nepal, Dhulikhel Hospital, WACEM, Kathmandu University Hospital, and other local stakeholder organizations.

### 2.3 Agenda

The conference was designed to meet the stated objectives which were achieved through a combination of focused half-day workshops, expert panel discussions with local and international leaders in disaster response, plenary sessions, logistics and planning meetings, and the TTX-based longitudinal workshop on disaster leadership and communication (Table 1). The TTX workshop was one component of the overall conference and was delivered in three distinct phases, each of which built on the lessons of the prior sessions but would also be meaningful as a stand-alone exercise.

**Table 1:** Conference agenda.

Day 1	Pre-conference workshops (participants had a choice to pick one of these, each had a cap of about 30 participants): Disaster Point of Care Ultrasound, Wilderness Medicine, CBRNE Decontamination, Tabletop Exercise curriculum, Pre-hospital care and hospital disaster preparedness planning
	Nepal leaders disaster response panel discussion
	Opening ceremony
Day 2	Essentials of critical care
	Plenary sessions
	International faculty disaster management experience sharing panel discussion
	Tabletop exercise curriculum
Day 3	Tabletop exercise curriculum
	Closing ceremony

The curriculum was adapted from previous TTXs conducted by this workgroup in various global contexts over the last decade, including India, Sri Lanka, Malaysia, South Africa, Qatar, and Turkey. The exercises introduced the topics of the incident command system (ICS), communication and leadership during mass casualty incidents (MCIs), developing hospital disaster preparedness protocols, and approach to managing chemical, biological, radiological, and nuclear (CBRN) incidents.

### 2.4 Curriculum development

The curriculum consisted of didactic lectures paired with a TTX (Table 2). The didactic lecture-based portions served as an introduction to key scientific theories and technical skills related to general disaster management topics. The paired TTXs aimed to teach the soft skills of leadership, communication, teamwork, situational awareness, and decision-making, where conference participants were challenged to troubleshoot locally adapted scenarios and apply the learned concepts. The educational content was delivered in English and participants were encouraged to communicate with each other and the facilitators in English. Exercises were developed based on guidelines provided by the HSEEP, incorporated concepts from the ICS framework, and followed a progressive approach whereby each exercise built on the concepts and objectives learned from prior exercises and increased in complexity (3).

**Table 2:** Tabletop exercise-based curriculum outline.

Day	Lecture topic	Tabletop exercise scenario	Conference objectives addressed
1	Incident command system	Terrorist threat at 2 mass gathering sites	1. Hospital ICS (HICS) implementation
	Approach to nuclear and radiological mass casualty incidents	Radiation detectors triggered on an arriving flight into the international airport	1. Technical knowledge and skills 2. Non-technical knowledge and skills 3. HICS implementation 4. Leadership skills 5. Patient and healthcare worker safety
2	Incident command system - refresher	Mass fatality incident involving a intercity bus accident	1. Technical knowledge and skills 2. Non-technical knowledge and skills 3. HICS implementation 4. Leadership skills 5. Patient and healthcare worker safety 6. Improve interhospital communication and coordination
	Mass fatality incidents		
	Psychological first aid		
3	Hazard vulnerability assessment	Earthquakes in several regions of the country prompting a national response	1. Technical knowledge and skills 2. Non-technical knowledge and skills 3. HICS implementation 4. Leadership skills 5. Patient and healthcare worker safety 6. Improve interhospital communication and coordination 7. Improve intra-departmental communication and coordination
	Incident response guides		
	Job action sheets		
	Disaster public messaging		

The specific curriculum topics were selected to provide a comprehensive yet general overview of key considerations in managing a disaster, and driven by the conference objectives of providing knowledge and skills for ICS implementation, improving disaster leadership, communication, and coordination skills, and addressing patient and healthcare safety concerns. While the specific scenarios were tailored to the local context, the topics covered were generalizable to any disaster. Focus was given on applying the roles and responsibilities of the ICS posts, developing a hospital response plan, managing mass casualties and fatalities, as well as patient surge, providing support for families and staff, and handling the psychological impacts of providing care during and after a disaster.

### 2.5 Exercise scenarios

The TTX scenarios were adapted to the local disaster governance framework, country geography, disease and disaster burden, health service capacity, and first responder and emergency medicine workforce structure, in an effort to simulate the existing conditions. This was done largely through review of literature on current national disaster framework policies in Nepal, as well as input from the faculty member who is Nepali and has extensive work experience in the country (10).

For the half-day workshop on the first day, the US-faculty led a group of participants through the basics of the ICS and management of radiological MCIs. One scenario for this topic was centered around a terrorist attack at two mass gathering sites, given the global impact of terrorism as well as the prevalence of mass gatherings around religious holidays in Nepal (14). A second radiological scenario was selected due to the rarity of radiological events in the country, under the assumption that it would be a topic participants would be least familiar with, and therefore fill a potential knowledge gap.

The second and third days of the conference brought together all the over 140 conference participants in a large auditorium. On the second day, the ICS was introduced again for the entire group to provide a standardized approach for organization. This was followed by lectures on mass fatality and psychological first aid. The lectures were followed by a TTX, where the main objective was to have participants plan their response utilizing ICS roles, coordinate among the different response entities involved, and anticipate health facility needs, including mass triage, patient transport, morgue capacity, and patient tracking. The TTX was based on an intercity bus accident scenario, given the high prevalence and healthcare burden of road traffic incidents in Nepal (13). Participants were broken up into groups representing a local health department, three hospitals with different resources and capacities, a first responder group, and a police group, and within each group assigned themselves ICS roles for the exercise. A specific adaptation was made in this exercise to account for a lack of a centralized pre-hospital ambulance system in the country, a role which private vehicles, taxis, and public transportation vehicles play (15, 16).

### 2.6 Model cities conception

The third and final day consisted of a culminating multi-city, multi-organizational exercise based on a model cities concept. Facilitators developed sample model cities of varying sizes based on the geography, demographics, and population of actual cities in Nepal. The model cities were based off of Kathmandu, Nepal’s capital and largest city, two smaller cities in less populated southern and western locales, and an outlying remote village in the far west of the country. Google Maps was used to estimate the distances between the model locales. While the general location in the country, size, population, demographics, and distances between each other were based on actual locales in Nepal, specific details regarding healthcare resources, physical infrastructure, industries, natural and constructed resources, and recent disasters were all created hypothetically to facilitate the flow of the exercise prompts and delivery of the workshop objectives. These key pieces of information allowed participants to put themselves into the simulation and think critically about how each locale’s hazards could affect the vulnerability and response to disasters.

Considering Nepal’s seismic activity, and at that time, the recent Jajarkot District earthquake, facilitators chose an earthquake scenario as it seemed like one of the most likely disaster scenarios that participants could face. A large magnitude earthquake would also require a national response in reality - allowing many different groups to be involved and, therefore, accommodating the large number of participants.

Participants were divided into equally-sized groups into the 4 locales, and then within each locale, were further organized into smaller sub-groups representing an appropriately leveled governmental emergency operations center, a hospital or clinic administration group, and hospital or clinic clinical staff group. Each group was provided information for their respective entity, a designed local map, and pertinent details about geography, distance in relation to the other cities, and their local hospital or clinic staffing and capabilities. These included number of beds, available specialty services, and number of operating rooms. An exact list of supplies was not provided. Participants were encouraged to simulate what resources would be available and what would need to be requested. In future simulations, this allows for participants to use their personal knowledge of the country to enhance the drill’s applicability. It also makes the plan more generalizable.

Armed with this information, facilitators instructed groups through the preparation phase of disaster response. Participants were guided through conducting a hazard vulnerability analysis for their assigned jurisdiction or facility, creating an incident response guide for an earthquake scenario, and filling out job action sheets for their respective ICS roles within their groups. Participants also had to develop memorandums of agreements with other cities and hospitals during this planning phase.

The following 2-h TTX required participants to apply the aggregate learned concepts from the 3 days to conduct a multi-city, multi-sector national earthquake response using the ICS framework and implementing and utilizing their incident response guides and memorandums of agreement. Prompts were given at certain times throughout the exercise to select groups who then had to respond and communicate the needs of their respective city and/or healthcare facility to the other groups in the room. This required participants to continually reassess and effectively communicate. These prompts let group members experience first-hand the challenges of disaster response. Debriefing sessions were conducted periodically to align all the groups together and learn from each other’s experiences. The number of prompts and number of debriefs that are performed during the tabletop can be adjusted to the needs and time-restraints of the group. Larger groups may need more prompts and debriefs to allow all participants to learn from each other, while smaller groups may be able to focus on a prompt for a longer period. This exercise stimulated participants to employ their leadership and delegation skills within their groups, troubleshoot inter and intra-group communication challenges, and work through addressing patients’ and healthcare providers’ health and safety needs during a disaster response event.

## 3 Discussion

### 3.1 Strengths

The host institute and organizing committee found the exercises particularly useful as knowledge and skill delivery methods. Given that there is limited knowledge on disaster management at the grassroots community level, the material from the exercises helped fill part of the national policy implementation gap for disaster response for healthcare workers.

Most of the participants were early career healthcare workers. In settings of MCIs, they would likely be on the frontlines of clinical care provision and less likely in administrative roles. Disaster resilience frameworks identify redundancy as one of the key components of disaster resilience and health systems strengthening (17, 18). A study looking at hospital resiliency at a large university hospital in Kathmandu after the 2015 earthquake revealed that redundancy and task-shifting played a major role in the immediate response (19). To this end, through the introduction of concepts and skills critical to managing disasters, the conference material contributed to resiliency building by providing a means of redundancy.

Through the TTX, an emphasis was placed on inter and intra-departmental and organizational communication. Participants were encouraged to troubleshoot patient care, transportation, supply-chain, and logistical issues within their assigned groups and with the other groups, mirroring the reality of the situations that arise in MCIs. As the final exercise progressed, facilitators were also able to teach the importance of communicating up the chain-of-command to effectively manage a disaster, and the fallouts that can occur when communication occurs in silos.

The TTXs used in this program as a training tool were adapted to the local context with the creation of model cities, to ease the participants’ understanding of the simulation and facilitate interactivity with the scenarios. The TTXs can be scaled to test disaster protocols at different levels, from the facility level all the way to a national level. The model cities concept that was used for the culminating exercise is especially useful for conducting an exercise aimed at coordinating a multi-agency multi-sector response.

### 3.2 Limitations

It is important to note that while this paper highlights the experiences and reflections from the perspectives of the organizers and facilitators, one limitation it has is lack of formal feedback from the participants. It would be beneficial to obtain evaluation data to help inform future exercises. Direct observation is one way to evaluate an exercise and requires assigned observers familiar with the exercise development, who observe each group involved and assess whether each group met the objectives, by what means, with how much input or guidance from a facilitator. In addition to director observation, participant feedback on the quality of the training sessions and faculty members, as well as a separate follow-up study to evaluate for content retention and application can help further augment future program organization and delivery. Additionally, while all participants spoke English, we did have Nepali speaking faculty who helped translate some points that were more complex. Using either live translators or having co-facilitators who speak the local language can be useful for capturing a wider audience in appropriate settings.

### 3.3 Looking ahead

Disaster response requires a multi-sector, multi-disciplinary approach with involvement from professionals and community members at various levels in the healthcare and community hierarchy. This conference hosted clinical staff, including physicians, nurses, and health assistants from around Nepal. Future conference efforts should focus on selectively involving a larger interdisciplinary participant base, including community health workers, medical personnel in supportive roles, police, staff from hospital clerical services and environmental services, public health professionals, and administrative leadership. Involving professionals from all involved sectors in preparedness efforts will only help augment coordination and communication during the response phase of the disaster cycle and set the stage for a unified response effort.

Through national legislation, Nepal has implemented a structure for disaster governance that mandates the establishment of emergency operation centers at the national, provincial, district, and local levels. While the framework provides a structure, studies cited above have found gaps in its implementation, a limited understanding of roles and responsibilities, inadequate training, and no standardized educational structure to deliver the training. This tabletop exercise-based workshop served as one means of filling this grassroots training and education gap. Further efforts must be made locally to sustain the momentum, build on the basic framework provided by such conferences, and expand it to include further protocol and policy development at the hospital level, with a multidisciplinary, multisector, whole community-involved training and education approach.

## Data availability statement

The original contributions presented in the study are included in the article/supplementary material, further inquiries can be directed to the corresponding author.

## Author contributions

JP: Writing – original draft, Writing – review & editing, Conceptualization. RK: Conceptualization, Writing – review & editing. JM: Conceptualization, Writing – review & editing. SB: Writing – review & editing. SS: Conceptualization, Writing – review & editing. BP: Writing – review & editing. BA: Conceptualization, Writing – review & editing, Supervision.
